# A Heuristic and Data Mining Model for Predicting Broiler House Environment Suitability

**DOI:** 10.3390/ani11102780

**Published:** 2021-09-24

**Authors:** Angel Antonio Gonzalez Martinez, Irenilza de Alencar Nääs, Thayla Morandi Ridolfi de Carvalho-Curi, Jair Minoro Abe, Nilsa Duarte da Silva Lima

**Affiliations:** 1 Graduate Program in Production Engineering, Universidade Paulista, R. Dr. Bacelar 1212, São Paulo 04026-002, Brazil; AAGMartinez@gmail.com (A.A.G.M.); jair.abe@docente.unip.br (J.M.A.); nilsasilvalima@gmail.com (N.D.d.S.L.); 2 Independent Researcher, Campinas 13100-650, Brazil; thaylamrcc@gmail.com

**Keywords:** broiler production, machine learning, random-tree, decision-tree, environmental temperature, ammonia concentration, relative humidity

## Abstract

**Simple Summary:**

The broiler housing control environment now is primarily based on the rearing temperature. The current study proposes two decision-tree models using flock-based and environmental data such as ambient temperature, air velocity, relative humidity, and ammonia concentration. Data from commercial broiler farms were collected and analyzed. An exploratory analysis employed the environmental variables, and a heuristic approach was used to develop a final dataset based on ammonia concentration’s impact on broiler production. The output models were related to dry bulb temperature, relative humidity, air velocity, and ammonia concentration arrays. The resulting trees classify the most suitable commercial broiler environment. Such variable combinations might help to improve environmental control in broiler houses.

**Abstract:**

The proper combination of environment and flock-based variables plays a critical role in broiler production. However, the housing environment control is mainly focused on temperature monitoring during the broiler growth process. The present study developed a novel predictive model to predict the broiler (*Gallus gallus domesticus*) rearing conditions’ suitability using a data-mining process centered on flock-based and environmental variables. Data were recorded inside four commercial controlled environment broiler houses. The data analysis was conducted in three steps. First, we performed an exploratory and descriptive analysis of the environmental data. In the second step, we labeled the target variable that led to a specific broiler-rearing scenario depending on the age of the birds, the environmental dry-bulb temperature and relative humidity, the ammonia concentration, and the ventilation rate. The output (final rearing condition) was discretized into four categories (‘Excellent’, ‘Good’, ‘Moderate’, and ‘Inappropriate’). In the third step, we used the dataset to develop tree models using the data-mining process. The random-tree model only presented accuracy for predicting the ‘Excellent’ and ‘Moderate’ rearing conditions. The decision-tree model had high accuracy and indicated that broiler age, relative humidity, and ammonia concentration play a critical role in proper rearing conditions. Using a large amount of data allows the data-mining approach to building up ‘if–then’ rules that indicate suitable environmental control decision-making by broiler farmers.

## 1. Introduction

Four main factors inside the housing environment affect the performance, productivity, and welfare of broiler chickens (*Gallus gallus domesticus*), the ambient dry-bulb temperature (T, °C), relative humidity (RH, %), ammonia concentration (NH_3_, mg·m^−3^), and the ambient air velocity (AV, m·s^−1^). Out of all the environmental parameters, dry-bulb temperature plays an essential role in maintaining body temperature within the normothermic ranges for mammals [1] and domestic fowl [2,3,4,5,6]. Therefore, most controlled environment systems rely solely upon this variable. 

The advised dry-bulb temperature for broilers in an initial stage inside the house is between 32.0 and 35.0 °C, decreasing 1.0 °C every two days until it reaches 24.0 °C [3,7,8]. For the third week (21 days), the temperature should be near 23.0 ± 2.0 °C [9,10,11]. Extreme heat or cold environments can harm the production cycle, not necessarily leading the bird to death [12,13]. Exposing young chicks to periods of long hours at a high temperature around 43 °C and limited access to water may result in dehydration, reduce the growth rate, and compromise broiler growth [4,14,15,16]. However, controlling the ambient temperature alone is not the best solution for reaching proper environmental control during the rearing of broiler chickens.

Ambient relative humidity also has an essential role in intensive animal production, affecting the thermal physical status, since the heat can be tolerable with a low humidity rate but is hardly bearable when it is high [17,18,19,20,21]. In a high humidity rate, during cold weather, wall condensation may occur inside the housing, increasing fermentation of the broiler litter, and diseases may occasionally appear in the flock [19,20,21,22,23,24].

Ammonia is a gas derived from uric acid microbial decomposition. The suggestion inside a broiler housing is to keep the concentration below 13.8 mg·m^−3^ [25]. The broiler chicken can easily catch respiratory diseases when the environmental ammonia is above 42.0 mg·m^−3^ [26,27]. If ammonia concentration reaches 69.4 mg·m^−3^, there is a decrease in the respiratory rate, which is harmful to the broiler’s physiological processes, negatively affecting performance [28,29,30].

The ventilation rate also has a direct effect on the performance of broilers [3,4,17,31]. A way of mitigating thermal stress is to properly interact air velocity and air temperature [18,32,33,34]. When the environment temperature is within the thermoneutral zone, broilers’ body temperature is kept at 42 °C [35], and when the environment temperature rises, biophysical processes are triggered. If these processes are not enough to keep homeothermy, the birds’ body temperature rises, leading to increased flock mortality by heat stress [24]. In the case of slaughter-age broilers, the environment temperature must be kept around 21 °C, the humidity must be kept near 50%, the air renewal might occur in less than 1.3 min, and the air velocity should be near 2.5 m·s^−1^ [2].

The environment inside the broiler house is a result of interconnected variables. A way of managing apparent cases of interlevel variables in a system is presented by Craver and Darden (2013) [36] that is applied in various areas of knowledge to develop models requiring a known input [37,38,39]. The algorithm first explores a set of data and looks for specific patterns to develop a model. The algorithm employs this evaluation’s results to define the mining model’s borders [40,41,42]. These boundaries are applied to the entire dataset for extracting possible patterns and detailed information [43,44]. 

Most environmental controllers in broiler houses use only the dry-bulb temperature as input, which leads to lessening the effect of the other environmental variables in the housing environment. The current study aimed to develop a novel predictive framework to forecast the broiler house suitability using a data-mining process centered on flock-based and environmental variables. 

## 2. Materials and Methods

### 2.1. Experimental Setup

**Figure 1:**
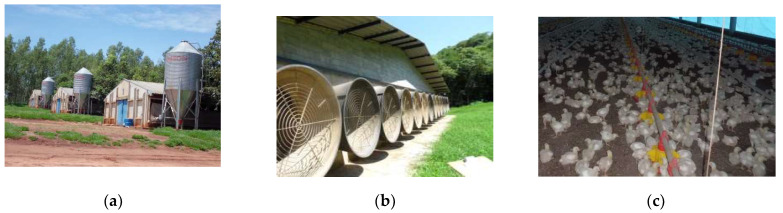
Overview of the commercial houses where the data were recorded (**a**), the air outlet fans (**b**), and inside the housing (**c**).

The research was conducted on a commercial broiler farm with four similar housing characteristics in Southeastern Brazil (latitude 22°42′04′′ S, longitude 46°45′52′′ W, altitude 674 m). The houses (20.0 m wide × 150.0 long × 2.9 m high) (Figure 1) had closed sidewalls with minimum ventilation outlets, with forced ventilation, ten exhausting fans (1 HP; 1.3 m ɸ; 38.000 m^3^ h^−1^), and sideways evaporative cooling panels (12.0 long × 1.80 m high, and 0.15 m wide cellulose board) to achieve environmental control. The roofs had fiber-cement tiles (14° angle) with a flat polypropylene material isolating the upper roof part. The front and rear buildings were solid brick walls, while the side walls had double blue polypropylene curtains. The automatic feeders provided feed, and water was given automatically using nipple drinkers. Flock density was kept in the range of 13–15 birds m^−2^. 

### 2.2. Variables Assessment

The environmental data (dry bulb temperature, °C; relative humidity, %; ammonia concentration, mg·m^−3^; air velocity, m·s^−1^) were recorded in three locations (air intake, center, and air exhaust) inside the houses when the broilers (Cobb^®^ 500) were 21, 28, 35, and 42-d-old. Dry-bulb temperature and relative humidity were continuously recorded using data loggers (RTR-507S, 0.1 °C, 1.5%, T&D, Matsumoto, Japan) in the houses and outside the buildings. The air velocity was registered using a hot wire anemometer (HWA2005DL, 0.2 to 20.0 m·s^−1^, 0.1 m·s^−1^, Long Branch, NJ, USA). Ammonia concentration data were recorded using an instantaneous gas concentration meter (GasAlertMicro 5, BW Technologies, Pittsburgh, PA, USA). The variables were recorded without interfering with the commercial broiler production. The birds were reared, complying with the breeder’s guidance.

### 2.3. Attributes, Target, and Data Analysis

The organized data consisted of a matrix A {A = (a*_ij_*), (*i*, *j* = 2496, 4)} containing the recorded values of the studied variables. There were four attributes and 2496 recorded instances. The data analysis was carried out in three steps. First, we used SAS^®^ software (SAS Institute Inc., Cary, NC, USA) to perform an exploratory and descriptive environmental data analysis. In a second step, we labeled the results into a range of values that lead to a specific broiler-rearing scenario depending on the age of birds, the environmental dry-bulb temperature and relative humidity, the ammonia concentration, and the ventilation rate. We used the environment variables thresholds suggested in the current literature (Table 1). We distributed the variables’ values from the references into a spreadsheet and calculated the quartiles’ median values. The values below the first quartile and above the upper quartile (lowest and highest 25% of numbers) were considered ‘Inadequate’. The median values were considered ‘Excellent’ (from 25.1% to 50%). The range between the median and upper quartile values was denoted as ‘Good’, and those between the median and highest distribution were considered ‘Moderate’. The output (rearing condition, RC) was then discretized (‘Excellent’, ‘Good’, ‘Moderate’, and ‘Inadequate’) according to the on-field results of the environmental variables. The RC ‘Excellent’ is the ideal environment where broilers achieve 100% of the performance established by the breeders, while ‘Inadequate’ means a housing condition where broiler performance is below 75%. The ‘Good’ and ‘Moderate’ conditions were distributed equally between the extremes. The third step is described further, and we built up a dataset using processed on-field recorded variables applied to a data-mining software.

To build up the variables array, we considered the distribution of a Markov process’s random conditions, finding the model to foresee the balance upon an interference using the initial conditions [46] and inferring multiple states to reach post-interference balance [47,48]. Each variable was given value at some time t + Δt as a function of the occurrence at time t (age, days; t ∈ {21; 42}) [49], where Δt is a fixed time interval (1 week = 7 days, starting at 21 days of growth). First, each environmental variable (dry-bulb temperature, relative humidity, and air velocity) was given an attributed weight as a function of the broiler’s age and considering the impact such a variable has on the rearing conditions using the suggestions given by the authors (Table 1). Such an array was used to build Table 2. The lower the broiler weight, the lower the variable’s impact in such a specific condition, leading to a decrease in broiler performance [21,22,27]. We used the values of Table 3 to estimate ARC values as a function of broilers age.

We assessed the initial RC using Equation (1).

(1) RC=(T∗WT)+(RH∗WRH)+(VA∗WVA)

where RC = rearing condition, T = dry-bulb temperature, WT = weight of temperature, RH = relative humidity, WRH = weight of relative humidity, VA = air velocity, and WVA = weight of air velocity. The equation was processed using standardized data from the on-field recorded variables.

For estimating the corrected rearing condition (ARC), which considers each variable’s weight in the overall housing environment, we added the impact of the ammonia concentration in broiler growth (Table 3). For this approach, we considered the detrimental effect of ammonia concentration inside the broiler house on broiler welfare and performance [2,17,26,27,45]. 

The corrected rearing conditions (ARC) used the ammonia concentration considering the following approach:

If NH_3_ = Excellent, then ARC = RC 

If NH_3_ = Good, then ARC = 0.75 ∗ RC 

If NH_3_ = Moderate, then ARC = 0.5 ∗ RC 

If NH_3_ = Inadequate, then ARC = 0.25 ∗ RC 

ARC is a deterministic value set between 0 and 10, corresponding to the rearing condition considering environmental variables, age, and ammonia concentration. We used a new set of rules to forecast the target (final rearing condition, ARCf) based on the following guidelines:

If ARC < 4, then ARCf = Inadequate

If 4 ≤ ARC < 6, then ARCf = Moderate

If 6 ≤ ARC < 8, then ARCf = Good

If ARC ≥ 8, then ARCf = Excellent

A software was developed to process the data and obtain the new values. The new dataset consisted of the recorded values and the studied variables’ processed discretized values [36].

**Figure 2:**
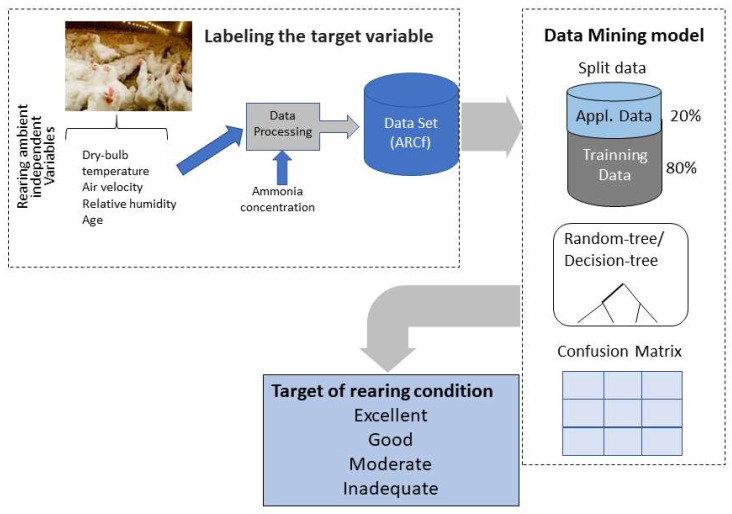
Schematic view of the data-mining model development and output.

The third step of the process was to build up a dataset using ARCf values. We applied the data-mining concept using the variables recorded in the field experiment (dry-bulb temperature, relative humidity, ammonia concentration, and air velocity). The target was the broiler ARCf. The Rapidminer^®^ Studio, a Java-based software version 9.2 (RapidMiner, Inc., Boston, MA, USA), was applied to process the data using the operators’ random-tree and decision-tree. We also pruned the resulting trees using the error-complexity pruning method [50] used by exploring the number of classification errors. The ‘decision-tree’ operator is a version of the C4.5 algorithm, one of the well-known decision-tree induction algorithms [51]. In the current study, we used 80% of the data to train the algorithm and 20% to develop the model. The data-mining model schematic is shown in Figure 2.

The confusion matrix was analyzed to find the accuracy (Equation (2)) using the classifying performance. The percentage of true positives (TP) to all positive predicted samples is the precision (Equation (3)), and recall is the ratio of correctly predicted positive observations to all observations in the actual level class (Equation (4)). Kappa statistic (κ) was used to check the instances classified by the data-mining model that matched the data labeled as accurate (how the expected accuracy measures a random classifier output). We accepted a model when κ > 0.60.

(2) $$\mathrm{Accuracy}\;\left(\%\right)=\frac{\mathrm{TP}+\mathrm{TN}}{\mathrm{TP}+\mathrm{FP}+\mathrm{FN}+\mathrm{TN}}$$

(3) $$\mathrm{Precision}=\frac{\mathrm{TP}}{\mathrm{TP}+\mathrm{FP}}$$

(4) $$R\mathrm{ecall}=\frac{\mathrm{TP}}{\mathrm{TP}+\mathrm{FN}}$$

where TP = true positive, TN = true negative, FP = false positive, TN = true negative, and FN = false negative.

## 3. Results

Table 4 shows the mean values and standard deviation of dry-bulb temperature, relative humidity, air velocity, and ammonia concentration for broilers’ age of 21, 28, 35, and 42 days. No significant variation was found in the rearing dry-bulb temperature, relative humidity, air velocity, or ammonia concentration. Such results were expected as the recorded values inside the controlled environment were based on dry-bulb temperature recommended by the breeders’ company. 

### Data Mining Models

**Figure 3:**
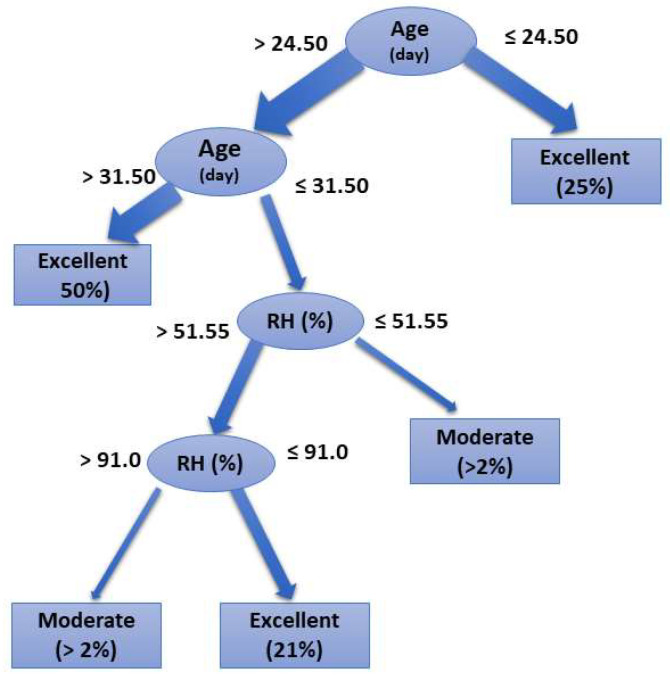
The random-tree operator’s result applied to the final rearing condition dataset, indicating that broiler age and relative humidity are determinants for selecting the rearing condition’s suitability.

The first data-mining model was performed based on the environmental thresholds, and the most suitable operator was a random-tree operator (Figure 3) with 89% accuracy (κ = 0.72). The result indicates that broiler age and RH are critical for the adequacy of the rearing environment. When ‘age’ is less than 24.50 days, then the ARCf is ‘Excellent’. When ‘age’ is higher than 24.50 days, then one must check on ‘RH’. If RH is higher to 91.0%, then ARCf is ‘Moderate’. If ‘RH’ is smaller or equal to 91.0%, then the ARCf is ‘Excellent’. 

The confusion matrix (Table 5) indicates that the random-tree operator provided an accurate classification (class precision) for 89% of the ‘Excellent’ results (*n* = 427) with a class recall of 97%. The ‘Moderate’ classification attended only a few samples (*n* = 10), indicating a low class recall (59%), quite similar to a randomized classification. The ‘Good’ and ‘Inadequate’ ratings were not detected in the samples (0%). Therefore, the random model is not adequate for predicting the broiler rearing environmental conditions that fall within this range.

**Figure 4:**
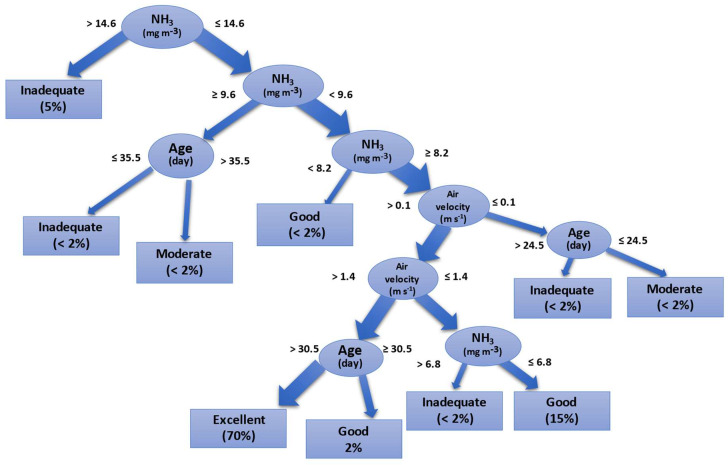
The decision-tree operator’s result applied to the final rearing condition, indicating that ammonia concentration is determinant for selecting the rearing condition’s suitability.

The second model was a decision-tree, with an accuracy of 99% (κ = 0.81). The decision-tree model is a tree structure that describes the classification of instances. The decision-tree consists of nodes and targeted edges. There are two types of nodes, internal nodes and leaf nodes. Internal nodes represent a feature or attribute, and leaf nodes represent a class. The decision-tree can be visualized as a set of ‘if–then’ rules. A rule is built from the root node of the decision-tree to each path of the leaf nodes. The characteristics of the internal nodes on the path relate to the conditions of the rules. The leaf nodes are associated with the classification of the targets. The found decision-tree initially used the variables NH_3_, air velocity, and broiler age to indicate the ARCf. The tree had presented six instances to predict 70% of the classification as ‘Excellent’. We applied the Error-Complexity pruning to reduce the leaves that led to the target that represented less than 1% of the samples, and the final decision-tree is shown in Figure 4.

The confusion matrix (Table 6) shows that the class precision and class recall of the condition ‘Excellent’ were adequate (99 and 100%, respectively). The conditions ‘Good’, ‘Inadequate’, and ‘Moderate’ were accurate but countered for a small sample (24, 22, and 16, respectively). 

## 4. Discussion

We aimed at developing a predictive model to forecast the broiler house suitability using a data-mining process centered on flock-based and environmental variables. The idea was to build a multi-variable model to serve as a basis for an optimized controlled environment system. The results showed two trees. One presented a random-tree with rules based on broiler age and the ambient relative humidity. However, it excluded dry-bulb temperature, ammonia concentration, and air velocity. The random trees classifier can handle a mix of categorical and numerical variables; however, the algorithm is less sensitive to data scaling [51]. Since we used weighted data considering the ammonia concentration, this probably affected the output. The second result was a decision-tree using the attributes ammonia concentration, age of broilers, and air velocity. Data were recorded in controlled temperature housing and maintained relatively constant according to the broilers’ age, as recommended by the breeders’ company. Therefore, we believe that such a condition led to the absence of the dry-bulb temperature attribute. 

According to the first tree, when broilers are younger than 24.5 days, RH needs to be evaluated. If RH ≤ 51.55%, then the ARCf is ‘Moderate’. If RH > 51.55%, then RH needs to be re-checked. If RH ≤ 91.00%, then the ARCf is ‘Excellent’. If RH > 91.00%, then the ARCf is ‘Moderate’. RH above 90.0% for an extended period might decrease productivity at any age [4,6], and market-size broilers are highly affected by long-time exposure to high RH [35]. This result reinforces RH’s importance inside broiler housing up to the fourth week of growth, primarily related to the minimum ventilation choice for young broilers [4,10]. Still, it lacks more detail for older broilers, mainly associated with the time of exposure to high values of RH at high temperatures. We recognize that it is nearly impossible to regulate relative humidity as precisely as temperature; however, efficient use of ventilation, as suggested by previous research [17], minimizes the impact of relative humidity higher than 75%. As the second tree result, the primary attribute for selecting the environmental rearing target was NH_3_. When NH_3_ > 14.6 mg·m^−3^, then ARCf is ‘Inadequate’. As for the results of NH_3_ ≤ 14.6 mg·m^−3^, the tree offers a range of options. We emphasize the branch representing 70% of the results (Figure 3) that indicate the ARCf is ‘Excellent’ when NH_3_ ≥ 8.2 mg·m^−3^, associated with air velocity > 1.4 m·s^−1^ and broiler age > 30.5 days. The other tree’ branches offer options for different settings, which might be found during broilers’ growth.

Air quality is a critical element in poultry production. The housing circulation air is the source of oxygen for the bird’s metabolism and a way for heat dissipation from water vapor, gases that come from the animals, manure decomposition, and dust released by the broiler litter [29,52,53]. The air quality must be determined through gas levels, dust, and microorganisms [27,30]. As the ammonia concentration builds up (because of the bird’s growth), the found decision-tree model indicates that the NH_3_ plays a critical role in the environment’s suitability. 

The importance of monitoring the air quality in broiler housings occurred to prevent health problems and meet animal welfare standards [33,54] due to labor health issues [30]. Previous research reports the interrelation between different ventilation systems, age, and flock density with ammonia build-up inside the housing [33,55]. Miragliotta et al. (2006) [56] found that the tunnel ventilation (AV > 1.8 m·s^−1^) managed to remove the gases inside the facilities with a density of 18 birds m^−2^, guaranteeing appropriate air quality. These previous results are comparable to the findings of the present study that used similar flock densities. Our decision-tree result showed that when the NH_3_ concentration is below 8.22 mg·m^−3^, AV is higher than 1.7 m·s^−1^, and the age is above 30.5 days, the rearing conditions are ‘Excellent.’ 

Ventilation is an effective way to reduce the dry-bulb temperature by convective heat loss [7,11]. It is also critical for production success, promoting humidity increase caused by the birds’ breathing, environment temperature control, environment oxygen renewal, and ammonia gas reduction [34,57]. Being a continuous procedure, the excess or lack of ventilation may interfere in the broiler production process results [31,32,58]. The ventilation inside broiler houses also fluctuates according to the season [27,59,60]. When the ventilation rate increases, relative humidity tends to decrease, and ammonia concentration tends to drop [61]. High relative humidity associated with high dry-bulb temperature leads to heat stress [20]; this could signal our random-tree result, enhancing RH as an attribute that impacts the final suitable rearing condition. Age is also a critical factor when applying the proper ventilation rate [4]. The current study results (Figure 2 and Figure 3) suggest that age is a contingent factor when analyzing the suitability of rearing conditions, agreeing with previous findings [18,57]. 

The effect of climatic variations and extreme weather events have become a significant challenge in poultry production. Broiler farming output and energy use depend on climatic conditions such as temperature and relative humidity [16]. Foreseeing the rearing condition using a historic dataset might help farmers mitigate significant weather variations. Ammonia concentration plays a critical role in air quality as ventilation control is not a straightforward concept. Our results (Figure 3) indicate that NH_3_ > 14.9 mg·m^−3^ leads to an unsuitable rearing environment. When the ammonia concentration is high, it affects broilers’ welfare status [62] and might impact economic trade.

Using a data-mining approach for solving classification problems and predicting non-linear results is relatively new [43,44]. The method presented here allowed the prediction of broiler housing environment suitability, considering simple assessed variables. The developed model’s enhancement encourages controller systems designers to add other environmental variables to fit broilers’ needs when considering the already-used dry-bulb temperature control threshold. Although appropriate environmental control avoids the incidence of high environmental temperatures due to weather conditions during the broiler grow-out, this variable alone is not enough to provide proper broiler rearing ambience. Such an approach might help implement an environmental control system more appropriately to optimize broiler performance.

## 5. Conclusions

The current modeling data suggest that broiler rearing conditions’ suitability is causally related to birds’ age, ammonia concentration, and relative humidity in a controlled environment housing. We believe that using only the input of dry-bulb temperature for environmental control might not be sufficient to meet proper welfare and performance levels during broiler growth. The data-mining approach using a large amount of data allowed us to build up ‘if–then’ rules that enable appropriate environmental decision-making control in broiler housing.

## Figures and Tables

**Table 1:** Description of the literature reviewed that provided a basis for the environment variables’ thresholds used to build up the model.

Source	Variable			
	Dry-Bulb Temperature (°C)	Relative Humidity (%)	Ammonia (mg·m^−3^)	Air Velocity (m·s^−1^)
Charles and Payne (1966) [45]			X	
Yahav (2004) [2]	X	X	X	
Dozier et al. (2005) [4]	X	X		X
Razuki et al. (2011) [6]	X			
Menegali et al. (2010) [7]	X	X		X
Souza et al. (2010) [8]	X			X
Vigoderis et al. (2010) [10]	X			X
Giloh et al. (2012) [11]	X			
Donkoh (1989) [12]	X			
Stringhini et al. (2003) [13]	X			
Yakav et al. (2005) [15]	X	X		X
Nawab et al. (2018) [16]	X			
Weaver and Meijerhof (1991) [17]		X		X
Furtado et al. (2006) [19]	X	X		X
Xiong et al., 2017 [20]		X		
Zhou et al. (2019) [21]	X	X		
Baracho et al. (2018) [22]	X	X	X	X
Fidaros et al. (2018) [23]		X		
Baracho et al. (2019) [24]	X	X	X	X
Homidan et al. (2003) [25]			X	
Chen et al. (2017) [26]			X	
Sa et al. (2018) [27]			X	
Wang et al. (2010) [29]			X	
Gudev et al. (2011) [30]			X	
Xin et al. (1994) [31]				X
Bucklin et al. (2012) [32]				X
Zhang and Li (2015) [33]				X
Gillespie et al. (2017) [34]				X
Vale et al. (2008) [40]	X			

X marks the information the reference adds to the variable.

**Table 2:** The range of the environmental variables discretized as a function of broilers’ age, the estimated RC, and the weight the rearing condition infers in the final calculation.

	Age (Day)			
Variable	21	28	35	42
Dry-bulb temperature (°C)	10 ≤ T < 16;	10 ≤ T < 16;	10 ≤ T < 16;	10 ≤ T < 16;
	Inadequate;	Inadequate;	Inadequate;	Inadequate;
	1	1	1	1
	16 ≤ T < 18;	16 ≤ T < 17;	16 ≤ T < 17;	16 ≤ T < 17;
	Inadequate;	Moderate;	Moderate;	Moderate;
	1	3	3	4
	18 ≤ T < 24;	18 ≤ T < 24;	18 ≤ T < 24;	18 ≤ T < 24;
	Moderate;	Excellent;	Excellent;	Excellent;
	4	10	10	8
	25 ≤ T < 28;	25 ≤ T < 28;	25 ≤ T < 28;	25 ≤ T < 28;
	Excellent;	Good;	Good;	Moderate;
	6	5	5	4
	28 ≤ T < 39;	28 ≤ T < 39;	28 ≤ T < 39;	28 ≤ T < 39;
	Inadequate;	Excellent;	Inadequate;	Inadequate;
	1	1	1	1
Relative humidity (%)	30 ≤ RH < 40;	30 ≤ RH < 40;	30 ≤ RH < 40;	30 ≤ RH < 40;
	Inadequate;	Inadequate;	Inadequate;	Good;
	1	1	1	6
	40 ≤ RH < 60;	40 ≤ RH < 60;	40 ≤ RH < 60;	40 ≤ RH < 60;
	Moderate;	Moderate;	Moderate;	Excellent;
	4	4	6	9
	60 ≤ RH < 70;	60 ≤ RH < 70;	60 ≤ RH < 70;	60 ≤ RH < 70;
	Excellent;	Excellent;	Excellent;	Moderate;
	8	8	9	4
	70 ≤ RH < 80;	70 ≤ RH < 80;	70 ≤ RH < 80;	70 ≤ RH < 80;
	Good;	Good;	Good;	Inadequate;
	6	6	4	4
	80 ≤ RH < 90;	80 ≤ RH < 90;	80 ≤ RH < 90;	80 ≤ RH < 90;
	Good;	Moderate;	Inadequate;	Inadequate;
	6	4	1	1
Air velocity (m·s^−1^)	0.0 ≤ AV < 0.2;	0.0 ≤ AV < 0.5;	0.0 ≤ AV < 1.25;	0.0 ≤ AV < 1.25;
	Inadequate;	Moderate;	Inadequate;	Inadequate;
	1	3	1	1
	0.2 ≤ AV < 0.4;	0.5 ≤ AV < 0.8;	1.25 ≤ AV < 1.9;	1.25 ≤ AV < 1.9;
	Good;	Good	Good;	Moderate;
	6	6	6	4
	0.4 ≤ AV < 0.5;	0.8 ≤ AV < 0.9;	1.9 ≤ AV < 2.0;	1.9 ≤ AV < 2.0;
	Excellent;	Excellent;	Good;	Good;
	8	8	6	6
	0.5 ≤ AV < 0.6;	0.9 ≤ AV < 1.0;	2.0 ≤ AV < 2.5;	2.0 ≤ AV < 2.5;
	Excellent;	Excellent;	Good;	Good;
	8	8	5	7
	0.5 ≤ AV < 0.6;	1.0 ≤ AV < 1.2;	2.5 ≤ AV < 3.0;	2.5 ≤ AV < 3.0;
	Moderate;	Excellent;	Good;	Good;
	4	8	8	8
	AV ≥ 0.6	AV ≥ 1.2	AV ≥ 3.0	AV ≥ 3.0
	Inadequate;	Excellent;	Excellent;	Excellent;
	1	8	5	5

**Table 3:** Range of ammonia concentration (mg·m^−3^) and its impact in the rearing environment corresponding to the broiler age.

Variable	Age (Day)			
	21	28	35	42
Ammonia (mg·m^−3^)	NH^3^ < 4.2;	NH^3^ < 4.2;	NH^3^ < 13.0;	NH^3^ < 13.0;
	Excellent	Excellent	Excellent	Excellent
	4.2 ≥ NH^3^ < 13.0;	4.2 ≥ NH^3^ < 13.0;	13.0 ≥ NH^3^ < 8.3;	13.0 ≥ NH^3^ < 8.3;
	Good	Good	Good	Good
	13.0 ≥ NH^3^ < 8.3;	13.0 ≥ NH^3^ < 8.3;	8.3 ≥ NH^3^ < 11.8;	8.3 ≥ NH^3^ < 11.8;
	Moderate	Moderate	Moderate	Moderate
	NH^3^ ≥ 8.3;	NH^3^ ≥ 8.3;	NH^3^ ≥ 11.8;	NH^3^ ≥ 11.8;
	Inadequate	Inadequate	Inadequate	Inadequate

Adapted from [2,22,24,25,26,27,28,29,30].

**Table 4:** Mean values and standard deviation of dry-bulb temperature, relative humidity, air velocity, and ammonia concentration.

	Age (Day)							
Variable	21		28		35		42	
	Mean	SD	Mean	SD	Mean	SD	Mean	SD
T (°C)	25.3	3.4	25.6	2.7	25.2	1.9	24.2	2.7
RH (%)	63.1	12.2	69.8	14.5	67.8	12.0	70.6	12.3
AV (m·s^−1^)	0.5	0.4	0.8	0.5	1.0	0.4	1.0	0.5
NH_3_ (mg·m^−3^)	4.0	0.8	4.3	1.5	4.0	1.5	4.2	0.8

SD = standard deviation.

**Table 5:** Confusion matrix of the random-tree algorithm applied to the rearing environment dataset.

	True	Excellent	Good	Moderate	Inadequate	Class Precision (%)
Predicted						
Excellent		427	24	7	22	89
Good		0	0	0	0	0
Moderate		0	0	10	0	54
Inadequate		5	0	0	0	0
Class recall (%)		97	0	59	0	-

**Table 6:** Confusion matrix of the decision-tree algorithm applied to the flock-based variables dataset.

	True	Excellent	Good	Moderate	Inadequate	Class Precision (%)
Predicted						
Excellent		436	0	1	0	99
Good		0	24	0	0	100
Moderate		0	0	16	0	100
Inadequate		0	0	0	22	100
Class recall (%)		100	100	94	100	-

## Data Availability

Data are available upon request to the corresponding author.
